# Translating ENIGMA schizophrenia findings using the regional vulnerability index: Association with cognition, symptoms, and disease trajectory

**DOI:** 10.1002/hbm.25045

**Published:** 2020-05-28

**Authors:** Peter Kochunov, Fengmei Fan, Meghann C. Ryan, Kathryn S. Hatch, Shuping Tan, Neda Jahanshad, Paul M. Thompson, Theo G. M. van Erp, Jessica A. Turner, Shuo Chen, Xiaoming Du, Bhim Adhikari, Heather Bruce, Stephanie Hare, Eric Goldwaser, Mark Kvarta, Junchao Huang, Jinghui Tong, Yimin Cui, Baopeng Cao, Yunlong Tan, L. Elliot Hong

**Affiliations:** ^1^ Maryland Psychiatric Research Center, Department of Psychiatry University of Maryland School of Medicine Baltimore Maryland USA; ^2^ Beijing Huilongguan Hospital Peking University Huilongguan Clinical Medical School Beijing People's Republic of China; ^3^ Imaging Genetics Center Stevens Institute for Neuroimaging and Informatics, Keck School of Medicine of USC Los Angeles California USA; ^4^ Department of Psychiatry University of California Irvine Irvine California USA; ^5^ Department of Psychology and Neuroscience Institute Georgia State University Atlanta Georgia USA; ^6^ Department of Pharmacy Peking University First Hospital Beijing China

**Keywords:** ENIGMA, gray matter, regional vulnerability index, schizophrenia, white matter

## Abstract

Patients with schizophrenia have patterns of brain deficits including reduced cortical thickness, subcortical gray matter volumes, and cerebral white matter integrity. We proposed the regional vulnerability index (RVI) to translate the results of Enhancing Neuro Imaging Genetics Meta‐Analysis studies to the individual level. We calculated RVIs for cortical, subcortical, and white matter measurements and a multimodality RVI. We evaluated RVI as a measure sensitive to schizophrenia‐specific neuroanatomical deficits and symptoms and studied the timeline of deficit formations in: early (≤5 years since diagnosis, *N* = 45, age = 28.8 ± 8.5); intermediate (6–20 years, *N* = 30, age 43.3 ± 8.6); and chronic (21+ years, *N* = 44, age = 52.5 ± 5.2) patients and healthy controls (*N* = 76, age = 38.6 ± 12.4). All RVIs were significantly elevated in patients compared to controls, with the multimodal RVI showing the largest effect size, followed by cortical, white matter and subcortical RVIs (*d* = 1.57, 1.23, 1.09, and 0.61, all *p* < 10^−6^). Multimodal RVI was significantly correlated with multiple cognitive variables including measures of visual learning, working memory and the total score of the MATRICS consensus cognitive battery, and with negative symptoms. The multimodality and white matter RVIs were significantly elevated in the intermediate and chronic versus early diagnosis group, consistent with ongoing progression. Cortical RVI was stable in the three disease‐duration groups, suggesting neurodevelopmental origins of cortical deficits. In summary, neuroanatomical deficits in schizophrenia affect the entire brain; the heterochronicity of their appearance indicates both the neurodevelopmental and progressive nature of this illness. These deficit patterns may be useful for early diagnosis and as quantitative targets for more effective treatment strategies aiming to alter these neuroanatomical deficit patterns.

## Graphical abstract

We developed the regional vulnerability index (RVI) to quantify individual similarity to the expected schizophrenia deficits patterns derived from large‐scale meta‐analyses performed by Enhancing Neuro Imaging Genetics Meta‐Analysis (ENIGMA) consortium. RVIs for cortical, subcortical, and white matter measurements and a cross‐modality RVI showed significant association with illness duration, cognitive deficits and symptoms. The similarity to expected disorder patterns captured by RVI may be useful for early diagnosis and as quantitative targets for more effective treatment strategies aiming to alter the formation of neuroanatomical deficits.

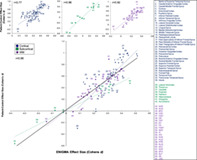

## 1 INTRODUCTION

The development of earlier and more effective therapies for schizophrenia has been hindered by the lack of robust brain biomarkers to identify the risks for developing this illness and to track its progression and response to treatment (Heresco‐Levy et al., 2002; Hoffman et al., 2003; Kane, Honigfeld, Singer, & Meltzer, 1988; Kulkarni et al., 2015; Samara et al., 2016). Large and inclusive Big Data meta‐analytic studies, such as these performed by the Enhancing Neuro Imaging Genetics Meta‐Analysis (ENIGMA) consortium show that patients with schizophrenia have reproducible and stable patterns of brain deficits including reduced cortical thickness, subcortical gray matter volumes and integrity of cerebral white matter (Kelly et al., 2018; van Erp et al., 2016; van Erp et al., 2018). We propose to translate the findings from these Big Data meta‐analyses to the individual level using a novel biomarker, the regional vulnerability index (RVI). RVI quantifies the similarity between an individual brain and the expected deficit patterns in schizophrenia derived from ENIGMA studies. The proposed biomarker can be derived using data within a single modality (unimodal RVI), or by combining data from multiple neuroimaging modalities (multimodal RVI).

The largest worldwide imaging studies in schizophrenia performed by the ENIGMA consortium offer a principled way to define disease‐related brain abnormalities with improved reproducibility (Kelly et al., 2018; Kochunov, Thompson, & Hong, 2019; van Erp et al., 2016; van Erp et al., 2018). The ENIGMA study of cortical thickness in schizophrenia (*N* = 4,474 patients; *N* = 5,098 controls) investigated 35 cortical regions (listed in supplementary Table S1) and found that patients had significantly lower average gray matter thickness, particularly in the frontal, temporal and parietal lobes (van Erp et al., 2018). In another study (*N* = 2,028 patients; *N* = 2,540 controls), the volumes of subcortical gray matter structures showed robust differences across cohorts (van Erp et al., 2016) including lower hippocampal volumes in schizophrenia patients compared to controls (Table S1). Finally, the ENIGMA study of white matter microstructural integrity in schizophrenia (*N* = 1,963 patients; *N* = 2,359 controls) used diffusion tensor imaging (DTI) derived fractional anisotropy (FA) maps of water diffusion (Beaulieu, 2002) to demonstrate widespread regional reduction in FA values in patients compared to controls (Kelly et al., 2018).

Findings in schizophrenia are commonly described using neurodevelopmental or neurodegenerative heuristics that are potentially conflicting (Kochunov & Hong, 2014). The neurodevelopmental and neurodegenerative alterations in the brains of schizophrenia patients may be manifested as an interaction between schizophrenia risk factors and lifelong trajectories for different brain compartments. In this study, we calculated RVIs for cortical, subcortical, and white matter compartments in early (≤5 years since onset), intermediate (6–20 years), and chronic (21+ years) stages of the illness to study the timing of the development of deficit patterns in different brain compartments.

The ENIGMA‐schizophrenia derived deficit patterns in cortical, subcortical and white matter measurements were found to be highly replicable in independent cohorts (*r* = .7–.9) (see Kochunov et al. 2020, this issue). This provides a strong rationale for translating these findings into measures of similarity between an individual and the expected disorder patterns. We propose the RVI as a simple correlational approach that uses ENIGMA‐derived deficits as the “ground truth” for expected regional schizophrenia‐related brain deficits. In prior work, we already showed the utility of the RVI approach by showing that unimodal, white matter RVI predicted treatment resistance in schizophrenia better than any individual tract‐specific white matter measures (Kochunov, Huang, et al., 2019). Therefore, we hypothesized that given the whole‐brain nature of schizophrenia a multimodal RVI may be more informative of the illness state than either unimodal RVIs or individual brain traits and would show stronger association with cognitive deficits and symptoms associated with this illness.

## 2 METHODS

### 2.1 Sample

We analyzed neuroimaging and clinical data from *N* = 122 patients and *N* = 78 healthy controls, the same sample previously used in the initial white matter RVI study (Kochunov, Huang, et al., 2019). Our analysis was limited to *N* = 120 patients (55M/65F, range = 17–65, average age = 38.3 ± 13.5 years) and *N* = 76 healthy controls (37M/39F, range = 17–65, average age = 38.6 ± 12.4 years) who had diffusion‐weighted imaging and T1‐weighted structural brain data that passed ENIGMA quality control (http://enigma.ini.usc.edu/protocols/imaging-protocols) (Stein, Medland et al., 2012). T1 data for four subjects failed quality control due to presents of motion and other artifacts. On average, the patients in this sample were diagnosed with schizophrenia for 16.3 ± 13.5 years (time since diagnosis = 0.1–42 years), which corresponded well with the three ENIGMA samples that provided cortical (10.5 ± 6.1 years, range = 0.6–20.2 years), subcortical (10.0 ± 5.7 years, range = 0.8–20 years) and white matter (14 years, range not specified) regional effect sizes (Kelly et al., 2018; van Erp et al., 2016; van Erp et al., 2018). Patients were further divided into three groups based on illness duration: early (within 5 years of diagnosis), intermediate (within 5–20 years of diagnosis), and chronic (over 20 years since diagnosis) (Table 1). We used the American Psychiatric Association guidelines to define the early group as patients who were within 5 years of the first episode of schizophrenia (Lehman, Lieberman et al., 2004). Chronic patients were defined as these who had schizophrenia for most of their adult life (>20 years with illness) (Lehman, Lieberman et al., 2004); the intermediate group captured patients between the two categories. All patients met DSM‐IV criteria for schizophrenia. Data were collected between 2017 and 2018. Patients were recruited from Beijing Huilongguan Hospital and controls through local advertisement; all had a homogeneous Chinese background. Exclusion criteria included current or past neurological conditions, unstable major medical conditions, and/or current or prior substance abuse (with the exception of nicotine/tobacco). All participants provided written informed consent according to the Helsinki Declaration, and the research protocol was approved by local Ethics Committees.

**TABLE 1:** Subject demographics for three patient groups and controls. Values are mean ± *SD*. Current mediation dose is expressed as CPZ equivalent units. All statistics were calculated using R software

	Controls (*N* = 76)	Early (*N* = 45)	Intermediate (*N* = 30)	Chronic (*N* = 45)	Analysis							
					Patients vs. controls		Early vs. intermediate		Early vs. chronic		Intermediate vs. chronic	
					*F*	*p*	*t*‐Score	*p*	*t*‐Score	*p*	*t*‐Score	*p*
*N* male/females	37/39	21/24	14/16	20/25	1.1	.7	0.8	.8	0.8	.9	1.1	.8
Age (years)	38.6 ± 12.4	28.4 ± 8.6	42.9 ± 9.4	51.7 ± 7.1	1.33	.25	6.89	1.60 × 10^−9^	13.98	3.62 × 10^−24^	4.61	1.62 × 10^−5^
PANSS (total)	—	70.3 ± 13.9	68.1 ± 23.1	67.9 ± 22.0	—	—	0.50	.62	0.61	.54	0.05	.96
Illness duration (years)	—	2.2 ± 1.1	14.8 ± 4.2	31.6 ± 6.5	—	—	19.01	3.33 × 10^−30^	29.76	4.9 × 10^−48^	12.49	6.68 × 10^−20^
Education (years)	13.2 ± 2.6	12.6 ± 3.4	12.3 ± 3.3	11.4 ± 2.7	6.76	.01	0.38	.71	1.77	.08	1.22	.23
Medication dose (CPZ)	—	327.5 ± 187.2	500.7 ± 246.1	596.2 ± 273.5	—	—	3.46	9.13 × 10^−4^	5.41	5.27 × 10^−7^	1.53	.13
Smoker^a^	24.6%	11.4%	36.7%	54.5%	1.64	.20	2.68	.009	4.79	6.75 × 10^−6^	1.52	.13

Abbreviations: CPZ, chlorpromazine; PANSS, Positive and Negative Syndrome Scale.

a Past and current smoking status.

Patient medication information, with chlorpromazine (CPZ) equivalent doses, is available in Table 1. Six patients were medication free at the time of study (early = 2/intermediate = 1/chronic = 3). Then, 7 (4/3/0) were taking first generation antipsychotics and 112 were taking the following second‐generation antipsychotics and others including: amisulpride, iloperidone, lurasidone, or quetiapine (1/4/4); 43 (4/7/34) patients were receiving multiple antipsychotic medications.

#### 2.1.1 Symptom and neurocognitive evaluations

Symptoms were evaluated using the Positive and Negative Syndrome Scale by one of three attending psychiatrists who maintained interrater reliability above 0.80. Cognitive function was assessed with the MATRICS Consensus Cognitive Battery (MCCB) which covered seven cognitive domains and yielded a composite score (Kern et al., 2008; Nuechterlein et al., 2008; Zou, Jiefeng, & Wang, 2009).

#### 2.1.2 Imaging protocol

Imaging data were collected using a 3T Prisma MRI scanner (Erlangen, Germany) at the Imaging Research Center of the Beijing Huilongguan Hospital, equipped with a 64‐channel RF head coil. Whole‐brain structural brain MRI data were acquired using a three‐dimensional‐magnetization prepared rapid acquisition gradient echo sequence with 0.8 mm isotropic resolution and the following sequence parameters: echo time (TE) = 2.2 ms; inversion time (TI) = 1,000 ms; repetition time (TR) = 2,400 ms; flip angle (FA) = 8°; field of view (FOV) = 256 mm × 256 mm. DTI data were collected using a spin‐echo, EPI sequence with a spatial resolution of 1.7 × 1.7 × 1.7 mm^3^. The sequence parameters were TE/TR = 87/8,000 ms; FOV = 200 mm; axial slice orientation with 82 slices and no gaps; 98 isotropically distributed diffusion‐weighted directions, two diffusion weighting values (*b* = 0 and 1,000 s/mm^2^); and five *b* = 0 images. Subjects' head movement was minimized with restraining padding.

### 2.2 Structural and DTI MRI data processing

All structural data were obtained using ENIGMA workflows that included quality control and assurance protocols (http://enigma.ini.usc.edu/protocols/imaging-protocols). In short, ENIGMA structural workflows are based on FreeSurfer (http://surfer.nmr.mgh.harvard.edu) and produce cortical gray matter thickness and subcortical volumes that are averaged across both hemispheres (regions listed in Table S1). The ENIGMA quality control assessment included visual inspection of each subject's external surface map and adjustment to the cortical ribbon; this step was performed by a neuroanatomist blinded to the diagnostic status. All regions of interest (ROIs) with a volume larger than 1.5 times the interquartile range were visually inspected by overlaying their segmentations on the subjects' anatomical images. These analyses yielded: average gray matter thickness measures for 35 cortical areas, the average whole‐brain cortical thickness (for the cortex overall), and the volumes of eight primary subcortical areas (region names listed in Table S1). DTI data were processed using the ENIGMA‐DTI analysis pipeline (https://www.nitrc.org/projects/enigma_dti) (Jahanshad et al., 2013). All data included in the analysis passed the ENIGMA‐DTI QA/QC protocol. Regional white matter FA maps were generated for 24 major white matter regions (listed in Table S1) based on the ENIGMA‐DTI atlas and averaged across hemispheres. The clinical data and the white matter RVI findings have been published (Kochunov, Huang, et al., 2019); the findings for cortical and subcortical RVI and the multimodality RVI approaches are new.

### 2.3 Statistics

#### 2.3.1 Regional vulnerability index

The ENIGMA schizophrenia consortium provided the meta‐analytical ranks of the severity of deficits associated with schizophrenia in gray matter thickness (35 cortical areas), subcortical volumes (8 structures), and FA (24 major white matter regions). These findings are provided as the regional effect sizes using Cohen's *d* statistics after adjusting for age and sex and for intracranial brain volume (Table S1). We developed the RVI as a simple measure of agreement between an individual's pattern of regional neuroimaging traits and the expected pattern of schizophrenia derived from ENIGMA meta‐analyses in these traits. The RVI is calculated as a single value per individual for patients and controls, and the average values for the controls are used to perform a z‐score normalization to achieve a contrast compatible with ENIGMA's *d*‐values. We further developed a multimodal RVI to derive a single index of agreement using data from multiple imaging modalities (described below). The calculations for the unimodal RVIs are as follows (using white matter RVI as an example): individual FA measures for each of the 24 major white matter tracts were converted to *z* scores in two steps: (a) We calculated the residual values by regressing out effects of the covariates used in the ENIGMA analyses including age, sex, and intracranial brain volume using the full sample (patients and controls), and (b) we performed a *z*‐score transformation for each individual/tract measurement using the average and *SD* values calculated in healthy controls. Specifically, for each subject, we subtracted the average value for a region and divided it by the *SD* calculated from the healthy controls. This produced a phenotypes vector of 24 z‐scores for regional white matter measurement for every individual in the sample. The RVI is then calculated as a Pearson correlation coefficient between 24 region‐wise *z* values for the subject and their corresponding effect sizes for schizophrenia‐control group differences in ENIGMA. This is repeated for gray matter thickness, which is represented by a vector of 35 values and subcortical gray matter volume, which is represented by a vector of eight values.

The multimodality RVI was calculated following a rank normalization of ENIGMA's Cohen's *d* values for the cortical, subcortical, and white matter measures by sorting and assigning values between 0 and 1, that is, the structure with the highest effect size had the value of 1 and the structure with the lowest effect size had a value of 0. This normalization maintained the relative distance between individual effect sizes within each modality and is intended to prevent biases during the multimodality RVI calculation due to potential differences in the magnitude of effect sizes across modalities. For example, it prevents the multimodality RVI coefficient from being exaggerated by any difference in the direction and the magnitude of effect sizes in different modality clusters. The multimodal RVI coefficient was calculated as a correlation between the full phenotypic vector of **57** values (35 cortical, 8 subcortical, and 24 white matter) for each individual and the corresponding vector of normalized ENIGMA effect sizes. Higher RVI values imply that the pattern of regional values more closely followed the regional vulnerability pattern for schizophrenia as determined by the ENIGMA meta‐analysis. The RVI calculator is distributed with the SOLAR‐Eclipse software (www.solar-eclipse-genetics.org).

#### 2.3.2 Group comparisons and correlation with cognition, symptoms, and current medication dose

All analyses were performed in R software version 3.6.0 (https://www.r-project.org) (R‐Development‐Core‐Team, 2009). A *t*‐test was used for whole‐sample patient‐control comparisons among demographic variables. Cohen's *d* effect sizes were calculated for patient‐control differences in individual brain traits after regressing age and sex effects. ANOVA was used to evaluate the significance of demographic variables among the three patient groups. ANCOVA was used to determine significant group differences for the regional cortical, subcortical, and white matter measurements, while controlling for age and sex. Bonferroni correction to reduce Type I errors associated with multiple comparisons set significance at *p* < .05/35 = 0.001 for cortical, *p* < .05/7 = 0.007 for subcortical, and *p* < .05/24 = 0.002 for white matter measures. All analyses first compared the combined patient group with controls.

Correlation analyses were used to study the associations among RVI, cognition and symptoms, and current medication dose. The correlation analysis between RVI and cognition was limited to the patients, to prevent overestimation of the association caused by patient‐control group differences in both RVI and cognition.

## 3 RESULTS

### 3.1 Patient‐control group differences in RVI

**FIGURE 1:**
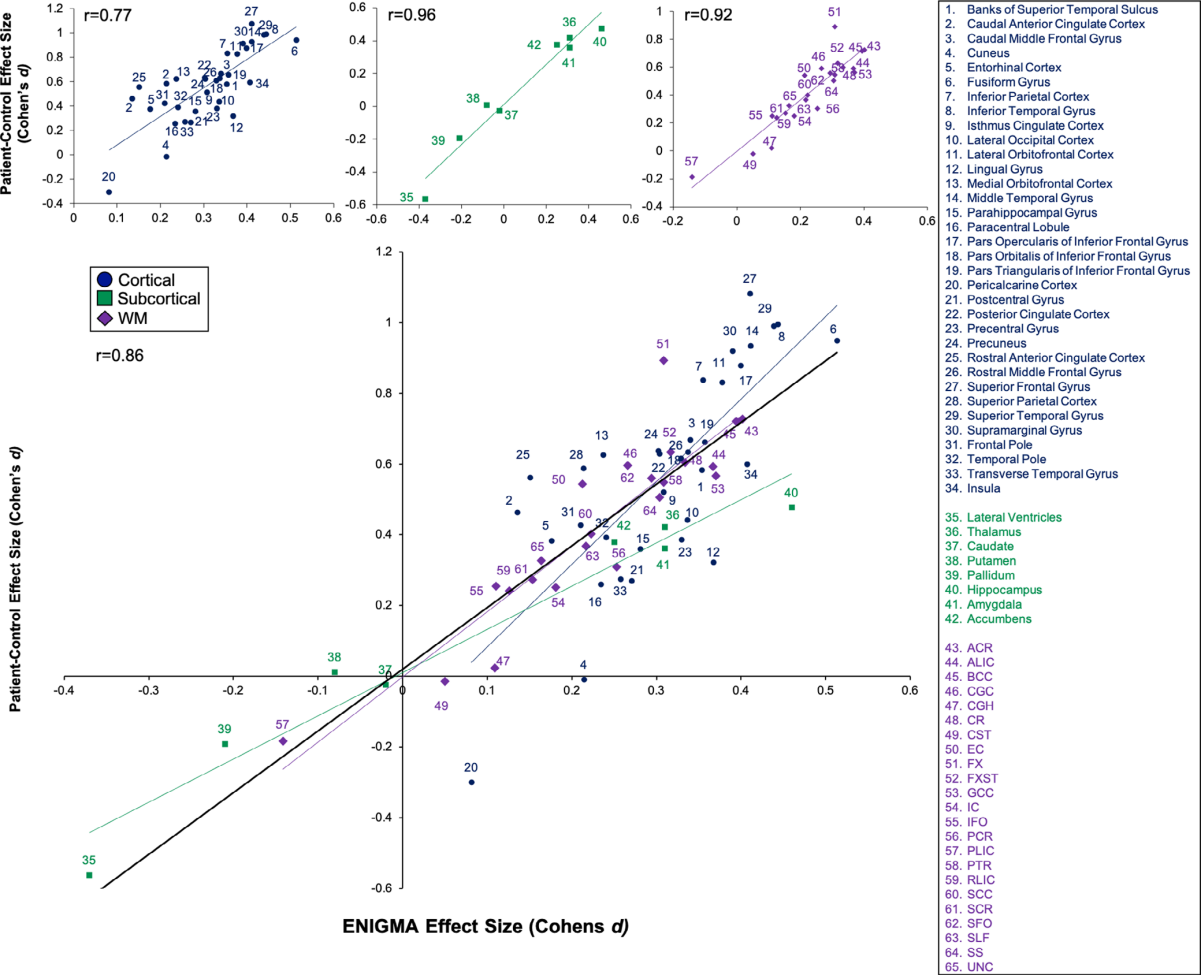
Scatterplot of the cortical, subcortical, and white matter effect sizes (Cohen's *d* coefficients, *y‐*axis) derived using the full patient‐control comparison in this sample versus the Enhancing Neuro Imaging Genetics Meta‐Analysis (ENIGMA) effect sizes (*x‐*axis) in the corresponding imaging modalities

Clinical characteristics of the sample are in Table 1. Patients showed widespread regional deficits across all three regional neuroanatomical measurement types (Table S1). The patient‐control group regional effect sizes for GM thickness, subcortical GM volumes, and white matter in this independent sample were significantly (*p* < 10^−5^) correlated with the respective effect sizes published by ENIGMA (Figure 1).

The highest correlation was observed for subcortical volume (*r* = .96), followed by white matter (*r* = .92) and cortical thickness (*r* = .77) deficit patterns (Figure 1). High correlations were likewise observed for deficit patterns in the early, intermediate and chronic groups (Figure S1, see supplement).

**FIGURE 2:**
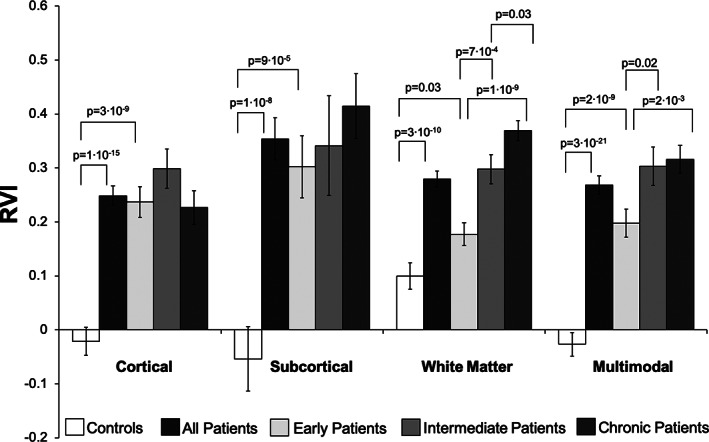
Group comparisons and *p*‐values for regional vulnerability index (RVI) measures. In general, the RVI, a measure of disease vulnerability or disease burden, increases as the disease progresses, but not for the cortical metrics

Patients showed significantly elevated RVI in the three modalities and the multimodality RVI compared to the controls (Figure 2): multimodal RVI showed the strongest effect size (Cohen's *d =* 1.57, *p* = 2.61 × 10^−21^) followed by cortical RVI (*d* = 1.23, *p* = 1.41 × 10^−15^), white matter RVI (*d =* 1.09, *p* = 3.31 × 10^−10^), and subcortical RVI (*d =* 0.61, *p* = 1.14 × 10^−8^ *)*. The effect sizes for each RVI were stronger than these derived from individual measurements in their respective domains. The difference among groups in individual measurements is provided in supplemental Tables S2–S5.

The RVI values in patients were significantly correlated for cortical and white matter (*r* = .28, *p* = .002), subcortical, and white matter (*r* = .23, *p* = .01) but not cortical and subcortical RVIs (*r* = .14, *p* = .3). There were no significant correlations among unimodal RVIs in controls (all *r* < .2, all *p* > .10).

### 3.2 RVI and cognition

The multimodal RVI showed a significant (after correcting for *N* = 28 comparisons) correlation with the total MCCB score that serves as the overall assessment of cognitive function (*r* = −.34, *p* = 10^−4^) (Table 2). In addition, the multimodal RVI was significantly correlated with the visual learning (*r* = −.33, *p* = 10^−4^) and working memory (*r* = −.30, *p* = .001) measurements (Table 2). It showed trending level significance with all other domains. Cortical and subcortical RVIs did not show significant correlation with any cognitive measurements after Bonferroni correction, with the exception of white‐matter RVI and processing speed (Table S6, see supplement).

**TABLE 2:** The correlation between the multimodality RVI and neurocognitive assessment scores

	Multimodality RVI	
	*r*	*p*
Visual learning	−.33	**1 × 10** ^**–4**^ ^a^
Verbal learning	−.20	*.03*
Social cognition	−.18	*.04*
Reasoning and problem solving	−.24	*.008*
Processing speed	−.25	*.005*
Working memory	−.30	**.001** ^a^
Total score	−.34	**1 × 10** ^**–4**^ ^a^

Abbreviation: RVI, regional vulnerability index.

a Significant correlations after correcting for *N* = 28 comparisons at *p* < .05/28 = .0017. *Italicized* values indicate nominally significant correlations (i.e., *p* < .05 but not passing the multiple comparisons correction).

### 3.3 RVI and clinical symptoms

White matter and multimodal RVI showed significant positive correlations with the severity of negative symptoms, after Bonferroni correction for *N* = 16 comparison (*r* = .36 and .27, *p* < .002, respectively) (Table 3). The correlation between negative symptoms and subcortical RVI was suggestive (*r* = .22, *p* = .02). There were no significant associations with either positive, global, or total symptoms for any RVIs.

**TABLE 3:** Correlations between PANSS symptom ratings and three types of vulnerability indices based on different sets of brain metrics and modalities, as well as a multimodality measure

PANSS symptoms	Cortical	Subcortical	White matter	Multimodality
Positive	0.04 (*p* = .65)	0.07 (*p* = .45)	−0.13 (*p* = .14)	−0.03 (*p* = .74)
Negative	0.07 (*p* = .44)	0.22 (*p* = .02)	0.36 (*p* = 5 × 10^−5^)^a^	0.27 (*p* = .002)^a^
Global	0.10 (*p* = .29)	0.06 (*p* = .54)	−0.12 (*p* = .19)	0.04 (*p* = .65)
Total	0.07 (*p* = .47)	0.13 (*p* = .15)	0.03 (*p* = .77)	0.07 (*p* = .46)

Abbreviation: PANSS, Positive and Negative Syndrome Scale.

a Significant correlations after correcting for *N* = 16 comparisons at *p* < .05/16 = .003.

### 3.4 RVI and illness duration

The RVI values for the early, intermediate, and chronic patient groups and healthy controls are shown in Figure 2 and Table 4. In the early illness stage, patients already showed significantly elevated cortical, subcortical, and multimodality RVIs compared to controls (*p* = 3 × 10^−9^, 9 × 10^−5^, and 2 × 10^−9^, respectively) but were not, on average, abnormally elevated for the white matter RVI (*p* = .03) after correction for *N* = 4 comparisons (Figure 2). Analysis of the four RVIs and three illness duration groups showed significant group effects for subcortical, white matter, and multimodal RVIs (*p* = .01, 10^−9^, .009). Post hoc analysis showed that there were no significant group differences for cortical or subcortical RVI across the three groups of patients (Figure 2). The white matter RVI showed significant stepwise increases from early to intermediate (*p* = 7 × 10^−4^), and from the intermediate to chronic (*p* = 1 × 10^−10^) groups (Figure 2). The multimodal RVI showed significant increases from early to intermediate (*p* = .02) but not from the intermediate to chronic stage (*p* = .76) (Figure 2).

**TABLE 4:** Effect sizes (Cohen's *d*) for RVI for early (up to 5 years since diagnosis), intermediate (6–20 years), and chronic (21+ years) patients versus controls

Patient groups	Cortical	Subcortical	White matter	Multimodality
Early	1.19 (*p* = 3 × 10^−9^)	0.46 (*p* = 9 × 10^−9^)	0.43 (*p* = .03)	1.27 (*p* = 2 × 10^−9^)
Intermediate	1.21 (*p* = 9 × 10^−10^)	0.83 (*p* = 5 × 10^−4^)	1.09 (*p* = 4 × 10^−6^)	1.79 (*p* = 2 × 10^−12^)
Chronic	1.16 (*p* = 3 × 10^−8^)	0.74 (*p* = 7 × 10^−7^)	1.55 (*p* = 9 × 10^−13^)	1.89 (*p* = 5 × 10^−17^)

Abbreviation: RVI, regional vulnerability index.

### 3.5 RVI, medication dose, and smoking

No RVI was significantly correlated with current medication dose (expressed as mg of CPZ, all *p* > .41) or smoking status (all *p* > .15) in the combined sample or any of the three patient groups.

## 4 DISCUSSION

We developed an RVI to quantify the similarity between regional patterns in an individual and the ENIGMA schizophrenia deficit patterns for: cortical gray matter thickness, subcortical gray matter volumes and white matter microstructural integrity as well as a multimodality index. All RVIs were significantly elevated in patients. RVI is calculated after correction for age and can be used to evaluate the impact of illness duration and convergence of individual toward expected schizophrenia deficit patterns. The cortical RVIs were highly elevated in the early diagnosis group compared to controls and remained stable with illness duration suggesting that these deficits had likely developed before the onset of illness. In contrast, the white matter and multimodal RVIs were higher in the intermediate and chronic versus the early group. We interpreted these findings by considering the impact of schizophrenia risk factors on the life‐long cerebral trajectory. Our findings suggest that the neuroanatomical deficits in schizophrenia affect the entire brain; the differences in RVIs with respect of illness duration suggest the life‐long nature of schizophrenia. Overall, the Big Data‐derived deficit patterns may be useful for developing novel biomarkers of early diagnosis and/or as quantitative targets for treatment strategies aiming to alter or prevent the formation of neuroanatomical deficits.

Historically, research in schizophrenia was characterized by high heterogeneity and poor reproducibility (Kochunov, Thompson, & Hong, 2019); however, big data research has greatly improved the stability of the neuroimaging, clinical, and genetic findings in this illness, see review by Kochunov et al., In press. The ENIGMA schizophrenia workgroup, in particular, has reported regional patterns of cortical, subcortical and white matter deficits in patients, by assembling the largest and most inclusive samples to date (Kelly et al., 2010; Kochunov, Thompson, & Hong, 2019; van Erp et al., 2016; van Erp et al., 2018). The deficit patterns derived by these studies predicted patient‐control differences in this (Figures 1 and S1) and other independent samples (Kochunov, Thompson, & Hong, 2019). This provided a strong rationale for developing measurements that gauge similarity between these patterns and those in an individual, such as the proposed RVI approach. The individual measurements of cortical thickness, subcortical volume, and white matter integrity are often analyzed as independent group contrasts. The RVIs are likewise individual level measurements; however, a sample of normal controls is used to achieve the contrast of group differences provided by ENIGMA. We demonstrated that the effect sizes for RVIs were stronger than those derived from individual trait values. The multimodality RVI showed the highest effect size among all measurements and demonstrated in advantage of aggregating phenotypes across diverse neuroimaging modalities into a meaningful index.

Multidomain neurocognitive deficits in schizophrenia are enduring, pervasive, and lead to functional disability in patients (Dickinson, Ramsey, & Gold, 2007; Faraone et al., 2000; Keefe et al., 2004; Keefe, Eesley, & Poe, 2005; Knowles, David, & Reichenberg, 2010). In patients, the multimodality RVI showed significant correlation with patients' scores on the visual learning and working memory domains and the total neurocognitive score and nominally significant suggestive correlations with all other domains. All correlations were negative, indicating that similarity with the schizophrenia was associated with worse performance across multiple cognitive domains. The highest correlation was observed between the multimodality RVI and composite MCCB total score. This supports the multimodality RVI construct and suggest that the composite MCCB score, the current “gold standard” for assessing cognitive deficits in schizophrenia, is sensitive to neurobiological deficits from diverse brain compartments and regions. As the patient's overall multimodality profile of brain metrics becomes more “schizophrenia‐like” (in the sense of being related to the ENIGMA schizophrenia pattern), the patient's cognitive deficit patterns may become more pervasive as captured by the MCCB total score.

The analysis of the early, intermediate, and chronic patient groups demonstrated differences among cortical, subcortical, white matter, and multimodal RVIs with respect to illness duration. The cortical deficit pattern as defined by RVI was already developed in the patients within 5 years of diagnosis and did not change with illness duration. In contrast, the multimodal and white matter RVIs were significantly higher in the chronic than in early diagnosis group suggesting ongoing illness progression. The subcortical RVI showed a step‐like pattern of increases among three groups but the differences were not significant (Figure S2). Such heterochronicity of the deficit patterns over time has led to potentially conflicting neurodevelopmental versus neurodegenerative heuristics in schizophrenia (Kochunov & Hong, 2014) with different models supported by different structural feature trajectories. RVI analyses allow us to explain this difference in trends by considering the heterochronicity of brain development; RVI measures may offer phenotypes for early diagnosis and research to prevent the worsening in the chronic stages of this illness.

Schizophrenia has been described using both neurodevelopmental and neurodegenerative heuristics. Neurodevelopmental heuristics, including the “two‐hit” hypothesis, suggest that the risk factors for this disorder impact early‐to‐adolescent development and then lead to the onset of psychosis with a stable disease course thereafter (Lewis & Levitt, 2002; Murray, O'Callaghan, Castle, & Lewis, 1992; Rapoport, Addington, Frangou, & Psych, 2005; Rapoport, Giedd, & Gogtay, 2012; Weinberger & Lipska, 1995). The findings of stable cortical RVI support this heuristic. It can be interpreted as evidence that that schizophrenia risk factors alter neuronal migration, synaptic reorganization, and pruning leading to a characteristic pattern of cortical thinning deficits prior to the onset of the illness.(Feinberg, 1982; Weinberger & Lipska, 1995). An alternative interpretation is that schizophrenia risk factors act continuously over lifespan and alter both cerebral development and aging. The peak of maturation of cerebral gray matter occurs around the age of puberty, and therefore, up to a decade before the average onset of psychosis. Therefore, the illness‐related alterations that led to a characteristic pattern of cortical deficits is expected to be present at the onset of the illness (Weinberger & Lipska, 1995). The cortical RVI may therefore present a valuable phenotype for early diagnosis of people at risk for schizophrenia and research in the prodromal stages of this illness.

White matter, multimodal and, to a lesser extent, subcortical RVI are supportive of the neurodegenerative heuristics in schizophrenia that began with Kraeplin's “*dementia praecox*” definition of schizophrenia as a progressive neurological disease with an elevated risk of dementia (Adityanjee, Aderibigbe, Theodoridis, & Vieweg, 1999; Anderson, O'Donnell, McCarley, & Shenton, 1998; Andreasen, 2010; Bleuler, 2010; Falkai et al., 2015; Jeste, Wolkowitz, & Palmer, 2011; Knoll et al., 1998; McGlashan, 2011). The findings of significant increases in the multimodal and especially white matter RVI in the intermediate and chronic patients appear to support these heuristics. However, the life‐long interaction between the risk factors and maturation and aging of cerebral white matter is a more likely explanation if we consider that the cerebral myelination continues into the fourth decade of life (Cancelliere et al., 2013; Gogtay et al., 2004; Kochunov et al., 2010; Kochunov, Glahn, Nichols, et al., 2011; Kochunov, Glahn, Lancaster, et al., 2011; Salthouse, 2009; Sowell et al., 2003). A large study that analyzed life‐long trajectory of white matter integrity in schizophrenia provide evidence to support this hypothesis by showing that peak myelination in patients occurs earlier (up to 5 years) than in controls and is followed by accelerated decline of the associative white matter tracts (Cetin‐Karayumak et al., 2019). This explains the relatively modest differences in white matter RVI for the early diagnosis group (average age = 28.5) as this age corresponds to the average age of peak for cerebral white matter in patients while controls continued to develop white matter into early fourth decade (Cetin‐Karayumak et al., 2019). Thus, the higher white matter RVIs in the intermediate and chronic groups are likely caused by earlier but smaller peak in myelination and then the detrimental interaction between schizophrenia risk factors and aging of cerebral white matter.

This study has limitations. It is based on cross‐sectional analyses and resolving the interaction between schizophrenia risk factors and neurodevelopmental and aging trajectories would require longitudinal data, especially in prodromal subjects and across a broad range of ages. The findings of significant correlation between RVI and neurocognitive and clinical data suggest that RVI may act *as* potential predictor at an individual level. However, a formal evaluation would require a longitudinal study that uses RVI as predictors of individual course. Our findings should encourage such studies. Patients in the intermediate and chronic groups had invariably longer exposure to medications and were more likely to be prescribed clozapine, compared to early stage patients. These factors could confound the discovery of the neurobiology of the illness by hindering the isolation of biomarkers from the medication effects. Ad hoc analyses did not show an association between RVI and the current dose of medication and did not show significant effects of clozapine treatment. However, longitudinal studies are required to test the association between RVI and cumulative medication dose in patients.

## 5 CONCLUSION

We used ENIGMA deficit patterns in schizophrenia to develop an RVI as a measure of similarity between the brain structural and microstructural patterns in an individual and the expected patterns in schizophrenia. We calculated RVI for three types of neuroimaging data, as well as a multimodality RVI, to evaluate anatomical, neurocognitive and symptomatic signatures at different stages of this illness. RVI may provide a valuable new phenotype of individual vulnerability, based on similarity to the expected patterns of disease progression rather than absolute difference between patients and controls. Similarity with the expected disorder patterns, quantified by RVIs, was associated with cognition, negative symptoms, and disease stages in an imaging domain specific pattern. Some deficit patterns were already established at early stages of diagnosis while others continue to develop with illness duration. We interpret this as a continuous life‐long interaction between risk factors for the illness and the normal trajectory of cerebral maturation and aging. RVIs may serve as a useful index for early diagnosis and prospective targets for therapies that aim to prevent or disrupt the processes of deficit pattern formation in this illness.

## CONFLICT OF INTERESTS

L. E. H. has received or plans to receive research funding or consulting fees on research projects from Mitsubishi, Your Energy Systems LLC, Neuralstem, Taisho, Heptares, Pfizer, Luye Pharma, Sound Pharma, Takeda, and Regeneron. None was involved in the design, analysis, or outcomes of the study. P. M. T. and N. J. received grant support from Biogen, Inc. (Boston, MA) for research unrelated to the topic of this manuscript. All other authors declare no conflicts of interest.

## AUTHOR CONTRIBUTIONS

Yunlong Tan, Peter Kochunov, and L. Elliot Hong had full access to the data and take responsibility for the integrity of the data and the accuracy of the data analysis. Peter Kochunov and L. Elliot Hong performed the analyses.

## Supporting information


**Appendix S1:** Supporting information

## Data Availability

Data will be made available by request through material sharing agreement.
