# Supplementary material for: Translating ENIGMA schizophrenia findings using the regional vulnerability index: Association with cognition, symptoms, and disease trajectory
Source: Hum Brain Mapp. 2020 May 28;43(1):566–75. doi: 10.1002/hbm.25045 (PMC8675428; doi:10.1002/hbm.25045)
Supplement: Supplementary file 1 — Appendix S1: Supporting information [file HBM-43-566-s001.docx]

| ***Cortical Thickness*** | **Controls** | **All Patients** | **Early**  **Patients** | **Intermediate**  **Patients** | **Chronic**  **Patients** | **All Patients vs. Controls Effect Size (Cohen's *d)*** | **P Value** |
| --- | --- | --- | --- | --- | --- | --- | --- |
| Average thickness | 2.63 ± 0.08 | 2.56 ± 0.09 | 2.61 ± 0.07 | 2.55 ± 0.08 | 2.52 ± 0.08 | 0.91 | **2.79x10^-9^** |
| Banks of Superior Temporal Sulcus | 2.64 ± 0.12 | 2.57 ± 0.13 | 2.64 ± 0.11 | 2.53 ± 0.14 | 2.53 ± 0.11 | 0.58 | **9.42x10^-5^** |
| Caudal Anterior Cingulate Cortex | 2.70 ± 0.19 | 2.59 ± 0.20 | 2.66 ± 0.17 | 2.60 ± 0.20 | 2.52 ± 0.20 | 0.46 | *1.78x10^-3^* |
| Caudal Middle Frontal Gyrus | 2.64 ± 0.12 | 2.57 ± 0.10 | 2.60 ± 0.10 | 2.55 ± 0.09 | 2.54 ± 0.11 | 0.67 | **8.64 x10^-6^** |
| Cuneus | 1.91 ± 0.12 | 1.91 ± 0.12 | 1.92 ± 0.13 | 1.92 ± 0.10 | 1.89 ± 0.11 | -0.01 | 0.95 |
| Entorhinal Cortex | 3.59 ± 0.24 | 3.50 ± 0.24 | 3.52 ± 0.28 | 3.50 ± 0.24 | 3.48 ± 0.20 | 0.38 | *9.71 x10^-3^* |
| Fusiform Gyrus | 2.87 ± 0.10 | 2.77 ± 0.12 | 2.82 ± 0.10 | 2.75 ± 0.12 | 2.73 ± 0.11 | 0.95 | **7.31x10^-10^** |
| Inferior Parietal Cortex | 2.59 ± 0.10 | 2.51 ± 0.10 | 2.55 ± 0.09 | 2.50 ± 0.09 | 2.47 ± 0.09 | 0.84 | **3.96 x10^-8^** |
| Inferior Temporal Gyrus | 2.91 ± 0.11 | 2.80 ± 0.11 | 2.83 ± 0.10 | 2.78 ± 0.12 | 2.78 ± 0.11 | 1.00 | **1.21x10^-10^** |
| Isthmus Cingulate Cortex | 2.37 ± 0.13 | 2.28 ± 0.17 | 2.34 ± 0.13 | 2.29 ± 0.18 | 2.21 ± 0.17 | 0.52 | **4.73x10^-4^** |
| Lateral Occipital Cortex | 2.32 ± 0.11 | 2.27 ± 0.10 | 2.28 ± 0.10 | 2.26 ± 0.10 | 2.25 ± 0.10 | 0.44 | 2.85 *x10^-3^* |
| Lateral Orbitofrontal Cortex | 2.70 ± 0.11 | 2.60 ± 0.11 | 2.66 ± 0.10 | 2.57 ± 0.10 | 2.57 ± 0.10 | 0.83 | **4.97x10^-8^** |
| Lingual Gyrus | 2.00 ± 0.12 | 1.98 ± 0.10 | 2.02 ± 0.09 | 1.98 ± 0.09 | 1.95 ± 0.10 | 0.32 | *0.03* |
| Medial Orbitofrontal Cortex | 2.58 ± 0.11 | 2.50 ± 0.13 | 2.56 ± 0.12 | 2.48 ± 0.12 | 2.46 ± 0.13 | 0.63 | **3.00x10^-5^** |
| Middle Temporal Gyrus | 2.97 ± 0.10 | 2.87 ± 0.12 | 2.94 ± 0.10 | 2.85 ± 0.12 | 2.81 ± 0.11 | 0.93 | **1.24x10^-9^** |
| Parahippocampal Gyrus | 2.69 ± 0.21 | 2.61 ± 0.23 | 2.69 ± 0.21 | 2.63 ± 0.22 | 2.50 ± 0.22 | 0.36 | *0.01* |
| Paracentral Lobule | 2.57 ± 0.13 | 2.54 ± 0.12 | 2.58 ± 0.10 | 2.52 ± 0.10 | 2.52 ± 0.13 | 0.26 | 0.08 |
| Pars Opercularis of Inferior Frontal Gyrus | 2.68 ± 0.12 | 2.58 ± 0.12 | 2.65 ± 0.10 | 2.56 ± 0.10 | 2.53 ± 0.12 | 0.88 | **9.17x10^-9^** |
| Pars Orbitalis of Inferior Frontal Gyrus | 2.73 ± 0.13 | 2.65 ± 0.15 | 2.70 ± 0.15 | 2.64 ± 0.12 | 2.59 ± 0.16 | 0.61 | **3.99x10^-5^** |
| Pars Triangularis of Inferior Frontal Gyrus | 2.52 ± 0.11 | 2.45 ± 0.11 | 2.51 ± 0.10 | 2.43 ± 0.09 | 2.41 ± 0.12 | 0.66 | **1.05x10^-5^** |
| Pericalcarine Cortex | 1.59 ± 0.13 | 1.63 ± 0.13 | 1.63 ± 0.14 | 1.64 ± 0.11 | 1.61 ± 0.12 | -0.30 | *0.04* |
| Postcentral Gyrus | 2.18 ± 0.10 | 2.16 ± 0.09 | 2.17 ± 0.09 | 2.16 ± 0.10 | 2.14 ± 0.09 | 0.27 | 0.07 |
| Posterior Cingulate Cortex | 2.54 ± 0.12 | 2.45 ± 0.14 | 2.53 ± 0.14 | 2.42 ± 0.11 | 2.40 ± 0.12 | 0.63 | **2.71x10^-5^** |
| Precentral Gyrus | 2.68 ± 0.12 | 2.64 ± 0.10 | 2.67 ± 0.10 | 2.63 ± 0.10 | 2.61 ± 0.10 | 0.39 | *8.86E-03* |
| Precuneus | 2.50 ± 0.10 | 2.43 ± 0.10 | 2.46 ± 0.10 | 2.41 ± 0.11 | 2.42 ± 0.10 | 0.64 | **2.11x10^-5^** |
| Rostral Anterior Cingulate Cortex | 2.90 ± 0.17 | 2.81 ± 0.17 | 2.89 ± 0.16 | 2.77 ± 0.14 | 2.74 ± 0.17 | 0.56 | **1.68x10^-4^** |
| Rostral Middle Frontal Gyrus | 2.43 ± 0.09 | 2.37 ± 0.09 | 2.40 ± 0.08 | 2.36 ± 0.08 | 2.35 ± 0.11 | 0.63 | **2.36x10^-5^** |
| Superior Frontal Gyrus | 2.82 ± 0.12 | 2.71 ± 0.10 | 2.77 ± 0.08 | 2.69 ± 0.09 | 2.67 ± 0.10 | 1.08 | **4.19x10^-12^** |
| Superior Parietal Cortex | 2.34 ± 0.10 | 2.29 ± 0.09 | 2.31 ± 0.09 | 2.28 ± 0.09 | 2.27 ± 0.09 | 0.59 | **8.48x10^-5^** |
| Superior Temporal Gyrus | 2.89 ± 0.13 | 2.77 ± 0.13 | 2.86 ± 0.11 | 2.74 ± 0.11 | 2.71 ± 0.11 | 0.99 | **1.51x10^-10^** |
| Supramarginal Gyrus | 2.65 ± 0.11 | 2.55 ± 0.11 | 2.59 ± 0.09 | 2.54 ± 0.12 | 2.51 ± 0.10 | 0.92 | **2.08x10^-9^** |
| Frontal Pole | 2.83 ± 0.17 | 2.74 ± 0.18 | 2.79 ± 0.17 | 2.74 ± 0.19 | 2.69 ± 0.18 | 0.43 | *3.90 x10^-3^* |
| Temporal Pole | 3.75 ± 0.25 | 3.65 ± 0.26 | 3.73 ± 0.28 | 3.63 ± 0.25 | 3.58 ± 0.22 | 0.39 | *7.76 x10^-3^* |
| Transverse Temporal Gyrus | 2.46 ± 0.17 | 2.41 ± 0.17 | 2.48 ± 0.15 | 2.36 ± 0.19 | 2.36 ± 0.15 | 0.27 | 0.06 |
| Insula | 3.01 ± 0.16 | 2.92 ± 0.16 | 3.01 ± 0.13 | 2.88 ± 0.13 | 2.85 ± 0.16 | 0.60 | **6.10x10^-5^** |
| ***Subcortical Volume*** | | | | | | | |
| Lateral-Ventricle | 14368.70 ± 7616.15 | 19928.62 ± 10650.47 | 14136.38 ± 5647.75 | 20295.69 ± 9828.99 | 25717.09 ± 12096.13 | -0.56 | **1.64x10^-4^** |
| Thalamus | 15494.92 ± 1840.79 | 14793.34 ± 1542.87 | 15427.75 ± 1437.79 | 14735.88 ± 1369.14 | 14172.17 ± 1538.35 | 0.42 | **4.43x10^-3^** |
| Caudate | 6925.30 ± 933.50 | 6901.05 ± 928.76 | 7116.23 ± 1040.47 | 6965.86 ± 805.59 | 6627.62 ± 837.58 | -0.02 | 0.87 |
| Putamen | 10218.11 ± 1312.75 | 10144.25 ± 1229.90 | 10403.31 ± 1253.67 | 10224.17 ± 1245.90 | 9813.66 ± 1142.34 | 0.01 | 0.93 |
| Pallidum | 4142.29 ± 445.91 | 4224.29 ± 460.08 | 4091.37 ± 460.37 | 4346.12 ± 476.39 | 4272.74 ± 421.39 | -0.19 | 0.19 |
| Hippocampus | 8180.09 ± 730.28 | 7785.38 ± 800.12 | 8113.58 ± 671.91 | 7661.74 ± 867.14 | 7533.92 ± 770.78 | 0.48 | **1.34x10^-3^** |
| Amygdala | 3444.64 ± 397.00 | 3291.34 ± 382.31 | 3408.60 ± 393.36 | 3260.24 ± 417.64 | 3191.77 ± 312.51 | 0.36 | *0.01* |
| Accumbens | 950.93 ± 175.68 | 885.47 ± 166.03 | 948.80 ± 164.43 | 873.02 ± 174.95 | 828.45 ± 139.43 | 0.38 | *0.01* |
| ***White Matter FA*** | | | | | | | |
| ACR | 0.444 ± 0.021 | 0.431 ± 0.028 | 0.445 ± 0.025 | 0.427 ± 0.023 | 0.419 ± 0.027 | 0.73 | **1.47x10^-6^** |
| ALIC | 0.569 ± 0.018 | 0.558 ± 0.024 | 0.568 ± 0.020 | 0.552 ± 0.025 | 0.553 ± 0.024 | 0.59 | **7.28x10^-5^** |
| Average FA | 0.356 ± 0.012 | 0.345 ± 0.014 | 0.352 ± 0.013 | 0.344 ± 0.014 | 0.340 ± 0.014 | 0.89 | **5.05x10^-9^** |
| BCC | 0.698 ± 0.032 | 0.677 ± 0.034 | 0.691 ± 0.025 | 0.668 ± 0.040 | 0.670 ± 0.033 | 0.72 | **1.87x10^-6^** |
| CC | 0.718 ± 0.023 | 0.703 ± 0.027 | 0.714 ± 0.021 | 0.697 ± 0.030 | 0.696 ± 0.028 | 0.62 | **3.16x10^-5^** |
| CGC | 0.639 ± 0.026 | 0.624 ± 0.029 | 0.629 ± 0.027 | 0.622 ± 0.030 | 0.620 ± 0.030 | 0.60 | **6.80x10^-5^** |
| CGH | 0.491 ± 0.035 | 0.489 ± 0.038 | 0.487 ± 0.038 | 0.488 ± 0.041 | 0.492 ± 0.038 | 0.02 | 0.87 |
| CR | 0.478 ± 0.017 | 0.469 ± 0.020 | 0.478 ± 0.020 | 0.466 ± 0.016 | 0.463 ± 0.019 | 0.60 | **5.17x10^-5^** |
| CST | 0.516 ± 0.024 | 0.514 ± 0.025 | 0.511 ± 0.025 | 0.518 ± 0.022 | 0.515 ± 0.027 | -0.01 | 0.92 |
| EC | 0.479 ± 0.016 | 0.471 ± 0.020 | 0.477 ± 0.019 | 0.468 ± 0.017 | 0.466 ± 0.020 | 0.54 | **2.61x10^-4^** |
| FX | 0.480 ± 0.036 | 0.448 ± 0.044 | 0.473 ± 0.037 | 0.446 ± 0.048 | 0.423 ± 0.035 | 0.89 | **5.41x10^-9^** |
| FXST | 0.540 ± 0.024 | 0.525 ± 0.031 | 0.537 ± 0.028 | 0.522 ± 0.028 | 0.515 ± 0.033 | 0.63 | **1.91x10^-5^** |
| GCC | 0.700 ± 0.024 | 0.687 ± 0.031 | 0.701 ± 0.026 | 0.679 ± 0.031 | 0.680 ± 0.031 | 0.57 | **1.53x10^-4^** |
| IC | 0.614 ± 0.015 | 0.609 ± 0.018 | 0.614 ± 0.017 | 0.606 ± 0.016 | 0.607 ± 0.020 | 0.25 | 0.09 |
| IFO | 0.517 ± 0.033 | 0.511 ± 0.035 | 0.508 ± 0.033 | 0.513 ± 0.032 | 0.512 ± 0.038 | 0.25 | 0.08 |
| PCR | 0.505 ± 0.021 | 0.499 ± 0.019 | 0.504 ± 0.020 | 0.499 ± 0.017 | 0.495 ± 0.020 | 0.31 | *0.04* |
| PLIC | 0.681 ± 0.019 | 0.683 ± 0.020 | 0.684 ± 0.021 | 0.680 ± 0.016 | 0.682 ± 0.022 | -0.18 | 0.21 |
| PTR | 0.588 ± 0.025 | 0.576 ± 0.029 | 0.585 ± 0.021 | 0.574 ± 0.025 | 0.567 ± 0.035 | 0.55 | **2.44x10^-4^** |
| RLIC | 0.580 ± 0.020 | 0.574 ± 0.021 | 0.577 ± 0.018 | 0.572 ± 0.021 | 0.573 ± 0.025 | 0.24 | 0.10 |
| SCC | 0.759 ± 0.019 | 0.751 ± 0.023 | 0.756 ± 0.019 | 0.751 ± 0.024 | 0.746 ± 0.026 | 0.40 | *6.51x10^-3^* |
| SCR | 0.502 ± 0.021 | 0.497 ± 0.021 | 0.502 ± 0.021 | 0.492 ± 0.017 | 0.496 ± 0.023 | 0.27 | 0.06 |
| SFO | 0.500 ± 0.025 | 0.487 ± 0.030 | 0.504 ± 0.026 | 0.476 ± 0.029 | 0.478 ± 0.028 | 0.56 | **1.56x10^-4^** |
| SLF | 0.500 ± 0.020 | 0.493 ± 0.022 | 0.494 ± 0.023 | 0.492 ± 0.017 | 0.493 ± 0.025 | 0.37 | *0.01* |
| SS | 0.524 ± 0.021 | 0.513 ± 0.026 | 0.520 ± 0.023 | 0.511 ± 0.022 | 0.508 ± 0.030 | 0.51 | **6.14x10^-4^** |
| UNC | 0.566 ± 0.042 | 0.554 ± 0.044 | 0.563 ± 0.040 | 0.563 ± 0.039 | 0.539 ± 0.047 | 0.33 | *0.03* |

**Table S1:** Average metric ± standard deviation for whole brain and regional gray matter thickness, gray matter subcortical volume, and white matter FA in this sample. Effect sizes (p-values) of illness for the regional measurements across three modalities performed in full patient-control comparison after correcting for age and sex. Effect sizes in individual patient groups were calculated versus the entire control sample after correcting for age and sex. **Bold** values indicate significance after correction for multiple comparisons; *italics* indicate nominal significance.

|  | **F-value for three group difference (p-values)** | **F-value for age effect (p-values)** | **F-value for sex effect (p-values)** |
| --- | --- | --- | --- |
| Average GM Thickness | *6.56 (0.01)* | *7.64 (0.007)* | 2.92 (0.09) |
| Banks of Superior Temporal Sulcus | 3.43 (0.07) | 3.28 (0.07) | 2.54 (0.11) |
| Caudal Anterior Cingulate Cortex | 1.83 (0.18) | 1.61 (0.21) | 0.23 (0.64) |
| Caudal Middle Frontal Gyrus | 2.09 (0.15) | *10.55 (0.002)* | 3.82 (0.05) |
| Cuneus | 0.51 (0.48) | 1.69 (0.20) | 0.34 (0.56) |
| Entorhinal Cortex | 1.42 (0.24) | 0.08 (0.77) | 1.14 (0.29) |
| Fusiform Gyrus | *4.75 (0.03)* | *5.11 (0.03)* | 1.93 (0.17) |
| Inferior Parietal Cortex | 2.93 (0.09) | 2.99 (0.09) | 2.34 (0.13) |
| Inferior Temporal Gyrus | 0.34 (0.56) | 1.10 (0.30) | 1.05 (0.31) |
| Isthmus Cingulate Cortex | 1.93 (0.17) | 0.20 (0.65) | 1.81 (0.18) |
| Lateral Occipital Cortex | 2.72 (0.10) | 0.71 (0.40) | 1.41 (0.24) |
| Lateral Orbitofrontal Cortex | 2.58 (0.11) | 3.61 (0.06) | 0.41 (0.52) |
| Lingual Gyrus | 1.26 (0.26) | 1.13 (0.29) | 1.40 (0.24) |
| Medial Orbitofrontal Cortex | 1.36 (0.25) | *5.77 (0.02)* | 0.30 (0.59) |
| Middle Temporal Gyrus | *5.68 (0.02)* | 1.99 (0.16) | 2.45 (0.12) |
| Parahippocampal Gyrus | 2.44 (0.12) | 0.01 (0.94) | 0.16 (0.69) |
| Paracentral Lobule | 2.25 (0.14) | *6.98 (0.009)* | 1.71 (0.19) |
| Pars Opercularis of Inferior Frontal Gyrus | *4.87 (0.03)* | *9.22 (0.003)* | *5.91 (0.02)* |
| Pars Orbitalis of Inferior Frontal Gyrus | 1.43 (0.23) | 1.32 (0.25) | 0.02 (0.89) |
| Pars Triangularis of Inferior Frontal Gyrus | *4.55 (0.03)* | *5.25 (0.02)* | 0.55 (0.46) |
| Pericalcarine Cortex | 0.23 (0.63) | 0.01 (0.92) | 1.44 (0.23) |
| Postcentral Gyrus | 0.87 (0.35) | 0.29 (0.59) | 1.91 (0.17) |
| Posterior Cingulate Cortex | *6.85 (0.01)* | 2.57 (0.11) | 0.40 (0.53) |
| Precentral Gyrus | 0.63 (0.43) | 2.92 (0.09) | 1.49 (0.22) |
| Precuneus | 1.17 (0.28) | 1.24 (0.27) | *4.72 (0.03)* |
| Rostral Anterior Cingulate Cortex | 2.61 (0.11) | *7.46 (0.007)* | 0.31 (0.58) |
| Rostral Middle Frontal Gyrus | 1.92 (0.17) | *8.87 (0.004)* | 1.13 (0.29) |
| Superior Frontal Gyrus | *6.24 (0.01)* | **22.76 (5.40x10^-6^)** | 0.64 (0.43) |
| Superior Parietal Cortex | *4.66 (0.03)* | *4.52 (0.04)* | 1.99 (0.16) |
| Superior Temporal Gyrus | *4.85 (0.03)* | *5.78 (0.02)* | 3.27 (0.07) |
| Supramarginal Gyrus | 3.91 (0.05) | *8.06 (0.005)* | 2.29 (0.13) |
| Frontal Pole | 1.17 (0.28) | *5.04 (0.03)* | 0.01 (0.91) |
| Temporal Pole | 3.37 (0.07) | 0.05 (0.83) | 0.59 (0.44) |
| Transverse Temporal Gyrus | 1.74 (0.19) | 0.81 (0.37) | 1.89 (0.17) |
| Insula | 1.86 (0.17) | 2.39 (0.12) | 3.02 (0.09) |

**Table S2:** Cortical gray matter across three patient group comparisons accounting for age and sex. **Bolded** values indicate significance after multiple comparisons correction (*p*<0.05/35=0.001); italicized values are nominally significant, with 0.05<*p*<0.001.

|  | **F-value for three group difference (p-values)** | **F-value for age effect**  **(p-values)** | **F-value for sex effect (p-values)** |
| --- | --- | --- | --- |
| Lateral Ventricle | *3.85 (0.05)* | 0.35 (0.55) | 0.65 (0.42) |
| Thalamus | 0.00 (0.95) | 0.46 (0.50) | 2.69 (0.10) |
| Caudate | 0.00 (0.99) | 0.00 (0.98) | *6.39 (0.01)* |
| Putamen | 0.77 (0.38) | 0.10 (0.75) | *4.61 (0.03)* |
| Pallidum | 2.79 (0.10) | 0.07 (0.79) | *4.78 (0.03)* |
| Hippocampus | 2.15 (0.15) | 0.01 (0.92) | 2.72 (0.10) |
| Amygdala | 0.72 (0.40) | 0.46 (0.50) | *7.27 (0.01)* |
| Accumbens | 0.08 (0.78) | 0.004 (0.95) | 1.77 (0.19) |

**Table S3:** Subcortical three group comparisons accounting for age and sex. Italicized 0.05<*p*<0.007.

|  | **F-value for three group difference (p-values)** | **F-value for age effect (p-values)** | **F-value for sex effect (p-values)** |
| --- | --- | --- | --- |
| ACR | **18.47 (3.61x10^-5^)** | *7.27 (0.008)* | 0.20 (0.66) |
| ALIC | **10.22 (0.002)** | *4.76 (0.03)* | 0.04 (0.84) |
| Average FA | **17.16 (6.56x10^-5^)** | *5.04 (0.03)* | 0.35 (0.56) |
| BCC | **11.20 (0.001)** | *3.97 (0.05)* | 0.12 (0.72) |
| CC | **8.39 (0.005)** | *4.75 (0.03)* | 0.25 (0.62) |
| CGC | 2.60 (0.11) | 1.82 (0.18) | 1.48 (0.23) |
| CGH | 0.01 (0.94) | 0.12 (0.72) | 0.17 (0.68) |
| CR | **12.57 (5.65x10^-4^)** | *3.98 (0.05)* | 0.30 (0.58) |
| CST | 0.005 (0.94) | 0.02 (0.88) | 1.37 (0.25) |
| EC | *5.53 (0.02)* | *4.16 (0.04)* | 0.03 (0.87) |
| FX | **36.36 (2.00x10^-8^)** | *6.06 (0.02)* | 0.05 (0.83) |
| FXST | **14.58 (2.17x10^-4^)** | 1.73 (0.19) | 0.05 (0.83) |
| GCC | **13.31 (3.97 x10^-4^)** | *8.95 (0.003)* | 0.06 (0.81) |
| IC | *4.75 (0.03)* | 0.64 (0.42) | 0.00 (0.95) |
| IFO | 0.07 (0.79) | 0.09 (0.76) | 0.03 (0.87) |
| PCR | 4.42 (0.04) | 0.17 (0.68) | 0.28 (0.60) |
| PLIC | 1.14 (0.29) | 0.30 (0.58) | 0.11 (0.74) |
| PTR | **16.68 (8.19 x10^-5^)** | 1.86 (0.17) | 0.01 (0.92) |
| RLIC | 1.67 (0.20) | 0.23 (0.63) | 0.00 (0.96) |
| SCC | *6.82 (0.01)* | 0.04 (0.85) | 1.28 (0.26) |
| SCR | *3.85 (0.05)* | 1.21 (0.27) | 0.01 (0.94) |
| SFO | **21.81 (8.16 x10^-6^)** | *7.03 (0.009)* | 0.60 (0.44) |
| SLF | 0.13 (0.72) | 0.42 (0.52) | 0.28 (0.60) |
| SS | *7.09 (0.009)* | 1.11 (0.29) | 0.13 (0.72) |
| UNC | *5.43 (0.02)* | 0.01 (0.94) | 0.04 (0.84) |

**Table S4:** White matter three patient group comparisons accounting for age and sex. Bolded values indicate significance (p<0.05/24=0.002); italicized 0.05<p<0.002.

| **MCCB Score Domain** | **Group** | **Age** | **Sex** |
| --- | --- | --- | --- |
| Visual Learning | 3.72 (0.06) | 0.07 (0.79) | 3.21 (0.08) |
| Verbal Learning | 2.65 (0.11) | 0.05 (0.82) | 0.41 (0.52) |
| Social Cognition | *6.91 (0.01)* | 0.67 (0.42) | 0.16 (0.69) |
| Problem Solving | 2.87 (0.09) | 0.04 (0.83) | 1.76 (0.19) |
| Processing Speed | *4.23 (0.04)* | 0.98 (0.32) | 0.87 (0.35) |
| Working Memory | *4.17 (0.05)* | 0.21 (0.64) | *4.25 (0.04)* |
| Total Score | *5.95 (0.02)* | 0.64 (0.43) | 2.58 (0.11) |

**Table S5:** Effect of patient group on neurocognitive tests after accounting for accounting for age and sex. Bolded values indicate significance (p<0.05/24=0.002); italicized 0.05<p<0.002

| **MCCB Score Domain** | **RVI** | | | | | | | |
| --- | --- | --- | --- | --- | --- | --- | --- | --- |
|  | **Cortical** | | | **Subcortical** | | | **White Matter** | |
|  | **r** | **p** | **r** | | **p** | **r** | | **p** |
| Visual Learning | -0.26 | *0.002* | -0.08 | | 0.35 | -0.20 | | 0.02 |
| Verbal Learning | -0.14 | 0.12 | -0.28 | | *0.002* | -0.08 | | 0.3 |
| Social Cognition | -0.05 | 0.6 | -0.13 | | 0.15 | -0.18 | | *0.04* |
| Reasoning and Problem Solving | -0.17 | 0.06 | -0.11 | | 0.18 | -0.14 | | 0.12 |
| Processing Speed | -0.14 | 0.13 | -0.21 | | *0.02* | -0.35 | | **1·10^-4*^** |
| Working Memory | -0.19 | *0.03* | -0.20 | | *0.03* | -0.11 | | 0.2 |
| Total Score | -0.23 | *0.01* | -0.20 | | *0.03* | -0.25 | | *0.006* |

**Table S6.** The correlation coefficients for modality specific RVI and MCCB scores. Bolded values indicate significance (after correcting for N=28, seven cognitive and four RVI, comparisons, p<0.05/28 = 0.001). *Italicized* values are suggestively significant correlation 0.001<p<0.05

**
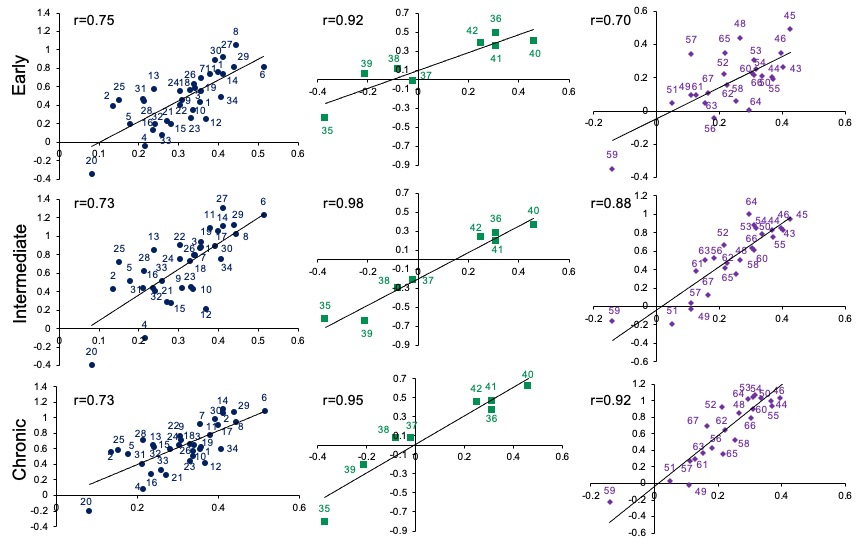
**

**Figure S1.** Correlation of patient-control effect sizes for three patient groups vs effect sizes provided by ENIGMA schizophrenia workgroup.


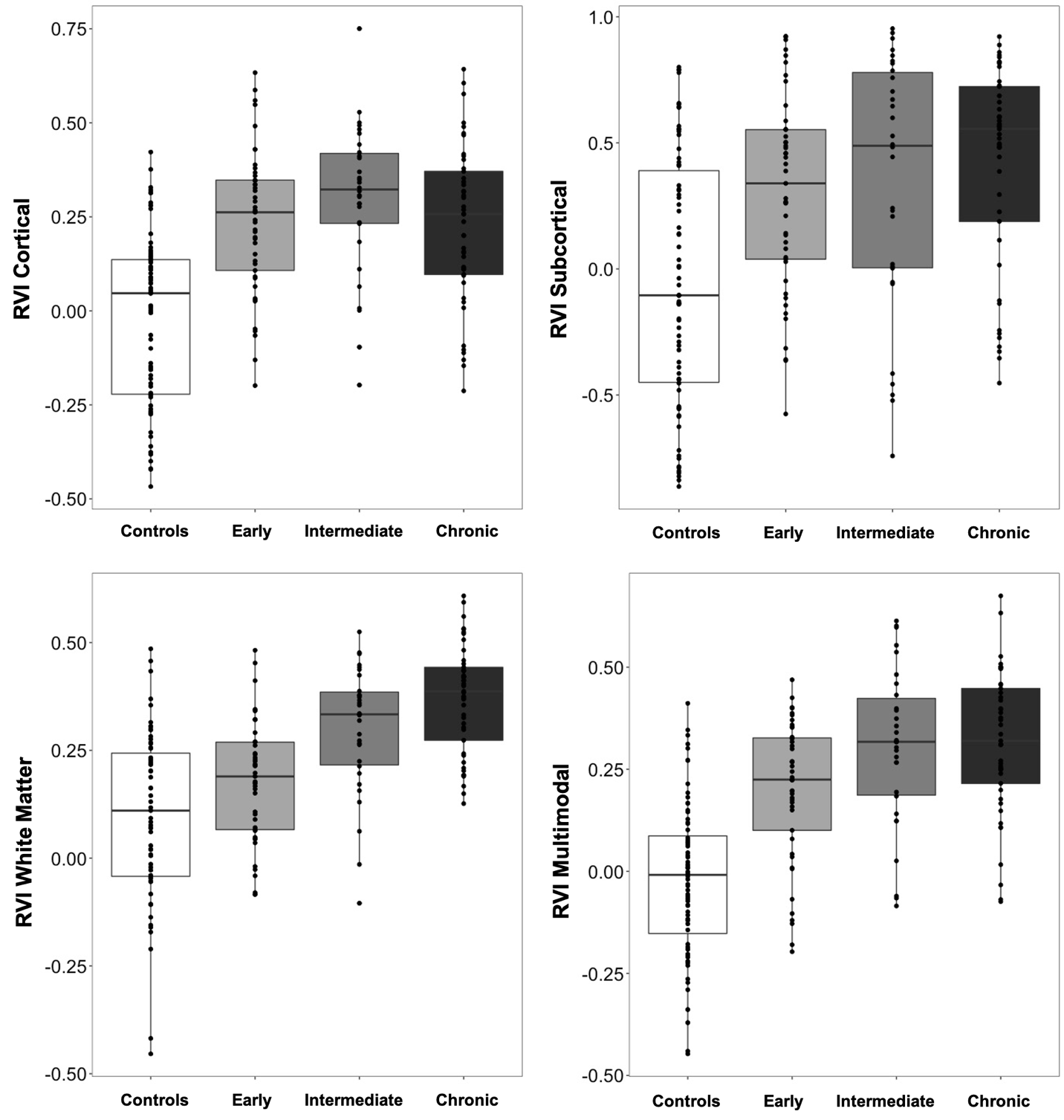


**Figure S2.** Box Plot of the Average RVI values in controls and the three patient group separated by illness duration: early (0-5 years), intermediate (6-20 years) and chronic (21+ years) groups.
